# Component Composition and Antimicrobial Activity of CO_2_ Extract of *Portulaca oleracea*, Growing in the Territory of Kazakhstan

**DOI:** 10.1155/2021/5434525

**Published:** 2021-01-22

**Authors:** Meruyert I. Tleubayeva, Ubaidilla M. Datkhayev, Mereke Alimzhanova, Margarita Yu Ishmuratova, Nadezhda V. Korotetskaya, Raisa M. Abdullabekova, Elena V. Flisyuk, Nadezhda G. Gemejiyeva

**Affiliations:** ^1^Department of Organization, Management and Economics of Pharmacy and Clinical Pharmacy, School of Pharmacy, Asfendiyarov Kazakh National Medical University, Almaty, St.Tole Bi, 88,050000, Kazakhstan; ^2^Department of Analytical, Colloidal Chemistry and Technology of Rare Elements, Al-Farabi Kazakh National University, Almaty, Al-Farabi Ave, 71, 050040, Kazakhstan; ^3^Department of Botany, E. Buketov Karaganda University, Karaganda, St. University, 28, 100028, Kazakhstan; ^4^Test Facility Management, JSC Scientific Center for Antiinfectious Drags, Almaty, Al-Farabi Ave, 75 A, 050060, Kazakhstan; ^5^Department of Pharmaceutical Disciplines and Chemistry, Medical University of Karaganda, Karaganda, St. Gogol, 40, 100008, Kazakhstan; ^6^Department Technology of Drags Forms, Saint-Petersburg State University of Chemical and Pharmaceuticals, St. Petersburg, St. Popova, 14, 197376, Russia; ^7^Laboratory of Plant Resources, Institute of Botany and Phyto-Introductions, Almaty, St. Timiryazev, 36 D, 050040, Kazakhstan

## Abstract

In the medicine of many countries, the use of herbal healing agents included a significant contribution to improving human health and well-being. Many antibiotics have been widely used to treat infectious diseases caused by various pathogenic bacteria. However, increased multidrug resistance has led to increased severity of diseases caused by bacterial pathogens. Bacteria remain the main causative agents of diseases that cause human death, even in the present day. This cause prompted scientists to investigate alternative new molecules against bacterial strains. The significant interest for the study is *Portulaca oleracea* L. (family *Portulacaceae*), a widespread annual plant used in folk medicine. Thus, the production and study of CO_2_ extract of *Portulaca oleracea* is an actual problem. *Methods*. Raw materials were collected from Almaty and Zhambyl regions (Southeast and South Kazakhstan) in phase flowering. *Portulaca oleracea* herb's CO_2_ extract was obtained by subcritical carbon dioxide extraction (installation of carbon dioxide flow-through extraction- 5L). The Wiley 7^th^ edition and NIST'02 library were used to identify the mass spectra obtained. The antimicrobial activity study was conducted by the micromethod of serial dilution and disco-diffuse method. Standard test strains of microorganisms were used: *Bacillus subtilis* ATCC 6633, *Staphylococcus aureus* ATCC 6538-P, *Candida albicans* ATCC 10231, and *Escherichia coli* ATCC 8739. *Results*. The use of carbon dioxide extraction (further CO_2_ extract) is a promising direction of obtaining total medicinal substances containing biologically active substances, from fractions of volatile esters of various composition and functional purpose until a fraction of fatty acids and fat-soluble vitamins. In the current study, we obtained CO_2_ extract at subcritical conditions from aboveground organs of *Portulaca oleracea* and investigated the component composition for the first time. From 41 to 66 components were identified in the composition of *Portulaca oleracea*‘s CO_2_ extract. Studies of antimicrobial activity showed that CO_2_ extract of *Portulaca oleracea* had the expressed effect against clinically significant microorganisms such as *Escherichia coli*, *Staphylococcus aureus*, *Bacillus subtilis*, and *Candida albicans*. *Conclusions*. This study showed that CO_2_ extract of *Portulaca oleracea*'s raw material contained biological active compounds exhibiting a significant antimicrobial effect.

## 1. Introduction

Plants from ancient times are a natural source of biologically active substances [1]. In the medicine of many countries, the use of herbal healing agents made a significant contribution to improving human health and well-being [2]. The World Health Organization (WHO) made a comprehensive analysis of the role of folk medicine in the world and published the “WHO Strategy in the field of folk medicine for 2014–2023” for integrating folk medicine into national health systems [3]. Medical preparations of plant origin are characterized by relative safety and low toxicity and act comprehensively on the human body, which allows applying them for the prevention and long-term treatment of diseases. Currently, more than 80% of the world's human population depends on herbal preparations for treatment of various human health problems [4].

Many antibiotics have been widely used to treat infectious diseases caused by various pathogenic bacteria. However, increased multidrug resistance has led to increased severity of diseases caused by bacterial pathogens. Thus, bacteria remain the main causative agents of diseases that cause human death even in the present day. The use of several antibacterial agents simultaneously (polypragmasy) in higher doses can cause toxicity for humans. This situation prompted scientists to investigate alternative new molecules against bacterial strains [5].

Conducting research on the introduction of plants with healing properties into official medicine is an actual problem; therefore, the use of local vegetative raw materials will increase production volumes and expand the range of medical preparations based on local plants.

The significant interest for the study is widespread annual plant *Portulaca oleracea* L. (family *Portulacaceae*), used in folk medicine. It vegetates from April to October; blooms from June to August; seeds mature from August to September. This species grows in gardens, on the melon fields, on streets settlements, in weed places, along the sandy coasts of reservoirs, and on roadsides, as a weed plant [6]. In Kazakhstan and CIS countries, it is successfully cultivated as ornamental and food culture [7].

Minh et al. report that the biologically active compounds, namely, flavonoids, alkaloids, fatty acids, terpenoids, sterols, phenolic compounds, proteins, and minerals, are present in *Portulaca oleracea* herb ethanolic and aqueous extracts [8]. The value of *Portulaca oleracea* is that it is a source of polyunsaturated fatty acids and antioxidants, which are necessary for maintaining human life [9].

Alcohol and aqueous extracts of *Portulaca oleracea*'s aerial part have a wide range of pharmacological properties, such as antioxidant, neuroprotective, anti-inflammatory, gastroprotective, hypoglycemic, hepatoprotective, antimicrobial, antipyretic, and antipyretic activities due to the content of various groups of biologically active compounds [10].

The authors of [11] studied polysaccharides from *Portulaca oleracea*, which have an antidiabetic activity, lowering blood glucose levels in alloxan-induced diabetic mice; in addition, the authors of [12] carried out studies where the polysaccharide component from this species exerts a pronounced antitumor effect on in vivo models.

The authors of [13, 14] present data on the biologically active compounds, namely, homoisoflavonoids portulaconones A-D and new alkaloid operaciamde C isolated from *Portulaca oleracea*'s extract that exhibits cytotoxic activity against four lines of human cancer cells and stem cells derived from human adipose tissue.

Scientific studies carried out in different years confirm the antioxidant activity of *Portulaca oleracea*'s methanol extract with the content of total phenols, flavonoids, carotenoids [15] and the phenolic compounds fraction from crude *Portulaca oleracea*'s extract [16].

The use of different extractants can affect the final content of biologically active compounds; the amount and composition of fatty acids were determined in the petroleum ether extract [17].

The use of carbon dioxide extraction is a promising direction for the production of total medicinal substances containing biologically active compounds, starting from volatile esters, fractions of various compositions, and functional purposes, ending with the fatty acids and fat-soluble vitamins fraction [18]. In this regard, the production and investigation of *Portulaca oleracea*'s CO_2_ extract is an urgent problem.

In the current study, we obtained the CO_2_ extract in the subcritical conditions from aboveground organs of *Portulaca oleracea* and studied the component composition and established the antimicrobial activity against pathogenic bacteria for the first time.

## 2. Materials and Methods

### 2.1. Sample Collection

The raw materials of *Portulaca oleracea* are collected in the flowering phase in 2-3 decades of August 2018-2019 in the foothill zone of Trans Ili Alatau (Almaty region, Southeast Kazakhstan) and in the floodplain of Talas River (Zhambyl region, South Kazakhstan). The raw material was harvested in dry weather. The drying of raw materials was carried out in a well-ventilated room at a temperature of +25 ± 5°C. The moisture content of the raw material should not exceed 10–12%. *Portulaca oleracea*'s raw material is stored at a temperature of +15°С–25°С and humidity of not more than 65%, in dry, well-ventilated rooms.

The plant samples were identified and transferred for storage to the herbarium fund of the Institute of Botany and Phyto-Introduction (Almaty city). The herbarium code of the sample of *Portulaca oleracea* is 2421/25, 2421/26.

### 2.2. Obtaining Carbon Dioxide Extract

*Portulaca oleracea* herb's CO_2_ extract was obtained from the aboveground part of the raw material in a laboratory facility for subcritical carbon dioxide extraction (installation of carbon dioxide flow-through extraction- 5L). The optimal conditions for obtaining CO_2_ extract were as follows: pressure 45–52, atmosphere, temperature +19–22°C, dynamic extraction time 540 minutes, and raw materials particle size 0.2–0.3 mm; the yield was 0.7%.

### 2.3. Component Composition Determination

The composition was determined on a gas chromatograph with an Agilent 6890N/5973N mass spectrometric detector. Chromatography conditions were as follows: sample volume 0.2 *μ*l and sample inlet temperature 240°C, without dividing the flow. The separation was carried out using a DB-35MS chromatography capillary column with a length of 30 m, an inner diameter of 0.25 mm, and a film thickness of 0.25 *μ*m at a constant carrier gas (helium) velocity of 1 ml/min. The chromatographic temperature was programmed from 40°C (holding 2 min) to 200°C with a heating rate of 10°C/min (holding 5 min) and up to 300°C with a heating rate of 20°C/min (holding 10 min). The detection was carried out in the SCANm/z 34–750 mode. Agilent MSD Chem Station software was used to control the gas chromatography system, recording, and processing the results and data.

### 2.4. Component Identification

The Wiley 7^th^ edition and NIST'02 library were used to identify the mass spectra obtained. The percentage of components was calculated automatically based on the peak areas of the total ion chromatogram. The components were identified by mass spectra and retention times.

### 2.5. Antimicrobial Activity Determination

To study the antimicrobial activity, standard test strains of microorganisms were used: *Bacillus subtilis* ATCC 6633 and *Staphylococcus aureus* ATCC 6538-P, which are obtained from the Republican Collection of Microorganisms (Nur-Sultan, Kazakhstan) and *Candida albicans* ATCC 10231 and *Escherichia coli* ATCC 8739, which are obtained from the American Type Culture Collection (ATCC, USA).

Sensitivity studies of microorganisms were performed on standard nutrient media:

  Mueller Hinton medium: Mueller Hinton Agar (М173), HiMedia, India; Mueller Hinton Broth (Mueller Hinton Broth (M391), HiMedia, India [CLSI]
  Fluid Sabouraud medium (M013), HiMedia, India [CLSI]

#### 2.5.1. Micromethod of Serial Dilutions

A 96-well plate was used to determine the antimicrobial activity [19, 20]. Mueller Hinton broth (for bacterial testing) and Sabouraud broth (for fungal testing) were introduced into the holes in an amount of 50 *μ*l. The extract was added in pure form in a volume of 50 *μ*l to the 1st and 2nd holes; starting from the 2nd hole, serial dilutions were prepared. The medium and test strain whole were used as a positive control to confirm growth for each test strain. A noninoculated hole containing nutrient broth without the test substance was used as a negative control for each test strain.

To all holes with dilution and positive control, 10 *μ*l of tested strain of the microorganism was introduced. The samples with bacteria were incubated at 36 ± 1°C for 24 hours. Samples with *Candida albicans* were incubated at 22 ± 1°C for 48 hours. The results were taken into account visually by the presence/absence of visible growth of test strains on the surface of the dense nutrient medium. The minimum bactericidal concentration (MBC) was considered the lowest concentration that suppressed microorganism growth.

#### 2.5.2. Disco-Diffuse Method

Suspension of microorganisms at a concentration of 1.5 × 10^8^ CFU/ml was seeded with a continuous uniform lawn on the entire surface of the Mueller Hinton agar [21, 22]. *Candida albicans* suspension at a concentration of 7.5 × 10^8^ CFU/ml was seeded with a continuous uniform lawn over the entire surface of the Sabouraud agar. It was held for 15 minutes, after which commercial discs, impregnated with the studied concentrations of extract, were applied to the surface of the inoculated culture and dried agar. Samples with bacteria were incubated at 36 ± 1°C for 24 hours and with *Candida albicans* were incubated at 22 ± 1°C for 48 hours. The results were taken into account by measuring growth suppression zones around the disks.

## 3. Results and Discussion

### 3.1. The Carbon Dioxide Extracts Component Composition

From 41 to 66 components were identified in the composition of *Portulaca oleracea*‘s CO_2_ extract (Tables 1–3).

Triterpenoids such as lupeol, *β*-amyrin, and *γ*-sitosterol; phytosterols such as campesterol and stigmasterol; diterpenes such as phytol; Vitamin E; monounsaturated fatty acids such as 9,12-octadecadienoic acid, ethyl ester, linoleic, ethyl linolenate, linoleic acid, methyl ester, and ethyl-9,12-octadecadienoate; polyunsaturated fatty acids such as linolenic acid and ethyl icosanoate; and fatty acids such as hexadecanoic acid, palmitic acid, methyl ester, palmitic acid, ethyl ester, and palmitic acid were found among the main groups of compounds for *Portulaca oleracea*'s CO_2_ extract.

## 4. Results of Antimicrobial Activity

Antimicrobial activity was studied on CO_2_ extract from the raw material of the Almaty region, Southeast Kazakhstan (2019), since the sum of terpenoids was 18.30% and fatty acids were 34.11%.

**Figure 1:**
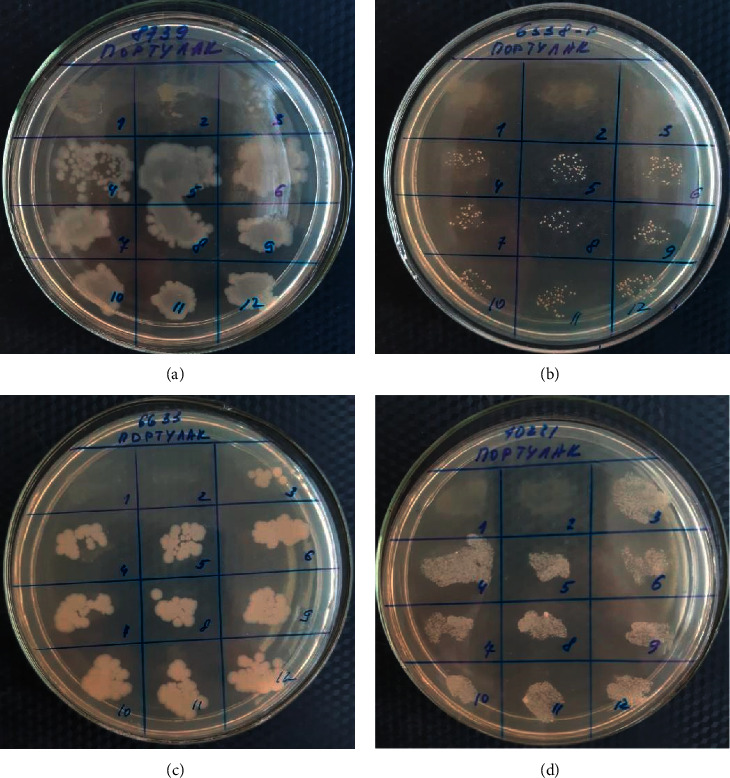
Results of the antimicrobial activity of *Portulaca oleracea*'s CO2 extract obtained by the serial dilution method from the Almaty region (Southeast Kazakhstan, 2019): (а) *E*. *coli*; (b) *S*. *aureus*; (c) *B*.*subtilis*; and (d) *C*. *albicans*. After studying the antimicrobial effect of *Portulaca oleracea*'s CO_2_ extract by the disco-diffuse method, its activity was also established. During interpreting the data, it was conditionally accepted that the diameter of the growth zone delay was over 15 mm- high activity, 10–15 mm- medium activity, and less than 10 mm- low activity (Table 5, Figure 2).

When determining the antimicrobial activity by the serial dilution method, the antibacterial and fungicidal activity of *Portulaca oleracea*'s CO_2_ extract was established in relation to analyzed strains of microorganisms *S*. *aureus*, *E*. *coli*, *B*. *subtilis*, *and C*. *albicans* (Table 4, Figure 1).

Previous studies confirmed the antimicrobial activity of *Portulaca oleracea*'s extracts. Thus, in the work of Chowdhary et al. [23], the antimicrobial activity of chloroform and ethanol extracts of *Portulaca oleracea* was reported via diffusion in agar against bacteria such as *Staphylococcus aureus*, *Bacillus cereus*, and *Klebsiella pneumonia* and fungi, as well as *Aspergillus fumigatus* and *Neurospora crassa*. The article by Zhou et al. [24] provided data on the antifungal and antibacterial activity of 70% methyl extract of *Portulaca oleracea* against *Candida albicans*, *Escherichia coli*, *Pseudomonas aeruginosa*, *Neisseria gonorrhoeae*, *Staphylococcus aureus*, *Bacillus subtilis*, and *Streptococcus faecalis*.

The observed results of our study are well consistent with the studies of Nayaka et al. [25], which reported that flavonoid apigenin isolated from the ethanol extract of the aboveground part of *Portulaca oleracea* showed an antibacterial activity on five pathogenic bacterial strains (*Pseudomonas aeruginosa*, *Salmonella typhimurium*, *Proteus mirabilis*, *Klebsiella pneumoniae*, and *Enterobacter aerogenes*) in in vitro experiments. Lei et al. [26] provided data on significant antibacterial effects of portulacebroside B, C, and D and portula ceramide isolated from *Portulaca oleracea*'s ethanol extract on enteropathogenic bacteria in in vitro experiments. In article of Syed et al. [27], an antifungal activity was detected in a sample of the plant *Portulaca oleracea* from Korea against some strains of the genera *Trichophyton* and significant broad-spectrum antibacterial activity against *Escherichia coli*, *Pseudomonas aeruginosa*, *Neisseria gonorrhoeae*, *Staphylococcus aureus*, *Bacillus subtilis*, and *Streptococcus faecalis*.

The results of the study of the antimicrobial activity by serial dilution showed that *Portulaca oleracea*'s CO_2_ extract had the greatest bactericidal effectiveness against *S*. *aureus* at the concentration of 250 *μ*g/ml; against *E*. *coli*, *B*.*subtilis*, and *C*. *albicans*, it has an established bactericidal activity at a concentration 500 *μ*g/ml.

When studying the effectiveness of *Portulaca oleracea*'s CO_2_ extract by the disco-diffuse method, data with high values of the growth suppression zone were also obtained, exceeding 15 mm. Thus, the growth retardation zone against *C*. *albicans*, *E*. *coli*, *S*. *aureus*, and *B*. *subtilis* was 15 mm, 18 mm, 20 mm, and 21 mm, respectively.

Extract of *Portulaca oleracea* from the Almaty region (Southeast Kazakhstan, 2019) has antimicrobial activity regardless of the research method.

Duarte et al. [28] and Galvao et al. [29] noted in their research that the herbal remedy was strong if it exhibited the antimicrobial effect at MBС (minimum bactericidal concentrations) values below 500 *μ*g/ml.

Thus, according to the results of the study, it was found that *Portulaca oleracea*'s CO_2_ extract has a pronounced antimicrobial effect.

## 5. Conclusions

The results of the study of the component composition of *Portulaca oleracea*'s CO_2_ extract obtained from raw materials of different origins are presented. The obtained extract identified 66 components from raw materials collected in the Zhambyl region and 41 and 50 components from raw materials collected in the Almaty region. The difference between the component compositions is explained by the soil climatic conditions of the regions. The main components in the raw materials are terpenoids, sterols, fatty acids, and tocopherols.

Study of the antimicrobial activity by serial dilution and the disco-diffuse method showed that *Portulaca oleracea*'s CO_2_ extract had a significant effect on the following microorganisms: *Escherichia coli*, *Staphylococcus aureus*, *Bacillus subtilis*, and *Candida albicans*.

## Figures and Tables

**Figure 2:**
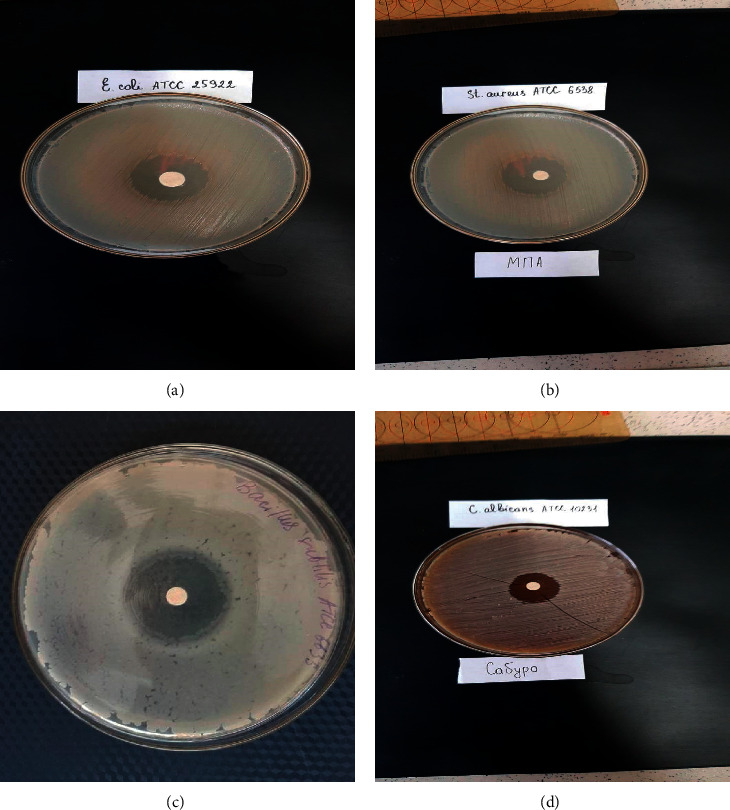
Results of the antimicrobial activity of *Portulaca oleracea*'s CO_2_ extract obtained by the disco-diffuse method from the Almaty region (Southeast Kazakhstan, 2019): (а) *E*. *coli*; (b) *S*. *aureus*; (c) *B subtillis*; and (d) *C*. *albicans*. *Portulaca oleracea*'s CO_2_ extract component composition varied according to the raw materials origin, place, and collection timing, which is explained by the difference in soil, climatic, and weather conditions. The chromatographic analysis sum of the main groups of compounds by classes is presented in Figure 3.

**Figure 3:**
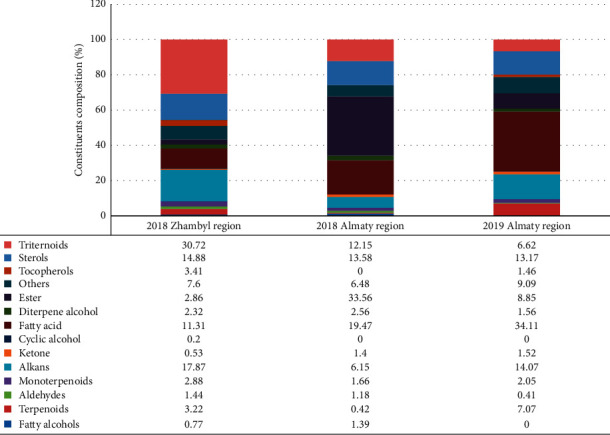
Ratio of main groups of substances in *Portulaca oleracea*'s CO_2_ extracts of different origin and time of raw material collection.

**Table 1:** The results of chromatographic analysis of *Portulaca oleracea*'s CO_2_ extract (Zhambyl region, South Kazakhstan, 2018).

No.	Retention time (min)	Compound	Identification probability (%)	Percentage (%)
1	10.2	2-Nonen-1-ol	79	0.15
2	12.5	Terpinen-4-ol	90	0.31
3	14.9	2-Decenal	87	0.12
4	15.6	Myrtenyl acetate	81	0.16
5	15.7	1-Undecene, 4-methyl-	80	0.47
6	15.9	2-Sec-butylcyclohexanone	75	0.15
7	17.0	2,4-Decadienal	91	1.00
8	18.3	Pentadecane	84	0.11
9	18.7	cis-*β*-Farnesene	81	0.14
10	18.8	1,3-Dioxane-5-methanol, 5-ethyl-	70	0.20
11	19.5	3-Cyclopenten-1-one, 2-hydroxy-3-(3-methyl-2-butenyl)-	75	0.23
12	20.8	Hexadecane	77	0.12
13	22.3	Dodecanoic acid	65	0.14
14	23.0	8-Heptadecene	79	0.09
15	23.2	trans-2-Dodecen-1-ol	80	0.41
16	23.5	Spathulenol	89	0.25
17	24.7	Loliolide	86	0.30
18	25.0	*α*-Bisabolol oxide B	92	0.46
19	25.3	*β*-Ionone, methyl-	70	0.09
20	25.4	Octadecane	78	0.21
21	26.6	Phytol	82	2.32
22	26.8	Myristic acid	93	1.29
23	27.6	Nonadecane	80	0.17
24	27.8	Bisabolol oxide A	87	0.53
25	27.8	Perhydro Farnesyl acetone	93	1.29
26	29.4	1-Dodecanol, 3,7,11-trimethyl-	73	0.21
27	29.6	Heptadecane	79	0.12
28	30.4	Herniarin	67	0.06
29	30.7	Phthalic acid, hex-3-yl isobutyl ester	90	0.26
30	30.9	Hexadecanoic acid	88	3.91
31	31.5	Heneicosane	89	0.23
32	32.8	1,6-Dioxaspiro[4.4]non-3-ene, 2-(2,4-hexadien ylidene)-	90	2.50
33	32.9	Dibutyl phthalate	90	0.24
34	33.4	Heptacosane	80	0.42
35	34.1	Ethyl oleate	79	0.21
36	34.4	9,12-Octadecadienoic acid, ethyl ester	85	1.53
37	34.6	Linoleic	88	4.46
38	34.7	Ethyl linolenate	78	0.87
39	35.0	Tetracosane	82	0.35
40	36.9	Docosane, 9-octyl-	72	0.19
41	37.6	Hexacosane	92	4.37
42	38.5	4,8,12,16-Tetramethylheptadecan-4-olide	89	0.71
43	38.6	Tetracosane, 11-decyl-	80	0.23
44	39.7	Oleyl oleate	65	0.20
45	40.0	Octacosane	81	4.91
46	40.1	Linolein, 2-mono-	72	0.27
47	40.2	Olein, 2-mono-	71	0.26
48	40.7	Butyl 9,12-octadecadienoate	77	0.78
49	41.0	Cannabidiol	77	0.20
50	41.7	Pentadecanal	78	0.31
51	42.1	Bis(2-ethylhexyl) phthalate	94	0.81
52	42.1	Tetratetracontane	72	1.41
53	42.5	2-Methyloctacosane	84	0.69
54	43.9	Tetradecyl acetate	92	1.44
55	44.4	Squalene	88	0.56
56	44.7	Hexacosyl acetate	83	1.04
57	47.3	1-Docosene	80	3.22
58	48.1	Vitamin E	71	3.41
59	50.4	Octadecyl trifluoroacetate	82	3.33
60	50.7	Campesterol	89	5.97
61	52.8	Stigmasterol	92	4.36
62	53.2	*γ*-Sitosterol	93	2.86
63	53.4	*β*-Amyrin	91	6.36
64	56.2	Lupeol	92	22.08
65	57.7	Simiarenol	77	2.27
66	58.0	Stigmast-4-en-3-one	81	1.70

**Table 2:** The results of chromatographic analysis of *Portulaca oleracea*'s CO_2_ extract (Almaty region, Southeast Kazakhstan, 2018).

No.	Retention time (min)	Compound	Identification probability (%)	Percentage (%)
1	17.0	2,4-Decadienal	91	0.77
2	22.5	Nonanoic acid, 9-oxo-, ethyl ester	87	0.63
3	23.2	Heptadecane	90	0.15
4	23.5	Spathulenol	87	0.15
5	24.7	Loliolide	88	0.42
6	25.4	Nonadecane	77	0.11
7	26.7	Tetradecanoic acid	93	1.26
8	26.9	Tetradecanoic acid, ethyl ester	89	0.61
9	27.8	2-Pentadecanone, 6,10,14-trimethyl-	91	1.40
10	29.7	Palmitic acid, methyl ester	92	0.53
11	30.9	Palmitic acid, ethyl ester	87	9.41
12	31.0	Palmitic acid	93	4.06
13	31.5	Heneicosane	90	0.21
14	32.9	Dibutyl phthalate	92	0.22
15	33.2	Phytol	79	2.56
16	33.3	Oleic acid, methyl ester	90	0.56
17	33.5	Linoleic acid, methyl ester	87	1.12
18	34.1	1,6-Dioxaspiro[4.4]non-3-ene, 2-(2,4-hexadien ylidene)-	90	0.86
19	34.5	Ethyl Oleate	91	3.40
20	34.6	Ethyl-9,12-octadecadienoate	89	10.84
21	34.8	9,12-Octadecadienoic acid	87	7.67
22	35.0	Linolenic acid, ethyl ester	81	5.65
23	35.3	Linolenic acid	90	6.48
24	38.1	Ethyl icosanoate	85	0.32
25	38.6	Hexacosane	91	1.27
26	38.7	4,8,12,16-Tetramethylheptadecan-4-olide	90	0.77
27	38.9	Octadecanal	74	0.41
28	41.2	Ethyl docosanoate	77	0.32
29	41.5	Docosyl acetate	90	0.54
30	41.7	Octacosane	92	3.74
31	42.1	Bis(2-ethylhexyl) phthalate	94	4.63
32	44.0	Tetratetracontane	87	0.67
33	44.2	Ethyl tetracosanoate	76	0.18
34	44.4	Tetracosyl acetate	92	0.97
35	48.1	Lignoceric alcohol	80	1.39
36	52.8	Campesterol	89	2.03
37	53.2	Stigmasterol	91	2.15
38	54.4	*γ*-Sitosterol	92	8.13
39	56.2	*β*-Amyrin	83	1.72
40	57.7	Lupeol	91	10.43
41	58.4	Stigmast-4-en-3-one	82	1.26

**Table 3:** The results of chromatographic analysis of *Portulaca oleracea*'s CO_2_ extract (Almaty region, Southeast Kazakhstan, 2019).

No.	Retention time (min)	Compound	Identification probability (%)	Percentage (%)
1	12.6	p-Menthan-3-one	91	0.23
2	14.9	Ethyl nonanoate	87	0.17
3	15.2	Pulegone	91	0.72
4	17.0	2,4-Decadienal	80	0.27
5	18.7	*β*-Famesene	93	1.91
6	20.3	*α*-Farnesene	86	0.13
7	23.5	Spathulenol	94	1.10
8	23.7	Caryophyllene oxide	85	0.15
9	24.0	Mint furanone	87	0.24
10	24.7	Loliolide	88	0.16
11	25.0	Bisabolol oxide II	93	2.60
12	25.7	*α*-Bisabolol	83	0.12
13	26.7	Myristic acid	90	0.55
14	26.9	Myristic acid, ethyl ester	83	0.29
15	27.6	2-Hexadecen-1-ol, 3,7,11,15-tetramethyl	77	0.15
16	27.8	Bisabolol oxide A	88	2.15
17	27.9	Hexahydrofarnesyl acetone	91	0.75
18	29.7	Benzoic acid, tridecyl ester	77	0.16
19	30.4	Herniarin	91	0.53
20	30.9	Hexadecanoic acid	84	10.07
21	32.8	1,6-Dioxaspiro[4.4]non-3-ene, 2-(2,4-hexadien ylidene)-	91	6.99
22	33.2	Phytol	94	1.56
23	33.9	7-Isopropyl-1,4-dimethyl-2-azulenol	71	0.42
24	34.5	Ethyl Oleate	91	1.35
25	34.6	Ethyl-9,12-octadecadienoate	88	6.74
26	34.8	9,12-Octadecadienoic acid	80	7.30
27	35.0	9,12,15-Octadecatrienoic acid, ethyl ester	95	4.67
28	35.2	9,12,15-Octadecatrienoic acid	84	9.20
29	36.4	Docosane, 7-hexyl-	87	0.66
30	37.9	Docosane, 11-butyl-	83	0.23
31	38.6	Hexacosane	93	4.39
32	38.7	4,8,12,16-Tetramethylheptadecan-4-olide	86	0.28
33	39.7	Tetracosane, 3-ethyl-	85	1.10
34	40.8	Olein, 2-mono-	72	0.27
35	41.5	1-Docosanol, acetate	91	0.73
36	42.7	Hexacosane, 9-octyl-	75	0.74
37	44.4	Tetracosyl acetate	91	0.87
38	44.6	Octacosane	93	4.14
39	44.8	Squalene	94	1.21
40	45.6	Tetratetracontane	76	0.30
41	47.2	Hexacosyl acetate	82	0.45
42	47.3	Hexacosane	89	1.29
43	48.1	Octadecyl Trifluoroacetate	78	0.99
44	50.5	Vitamin E	88	1.46
45	52.8	Campesterol	88	1.52
46	53.2	Stigmasterol	87	3.61
47	54.4	*γ*-Sitosterol	94	8.04
48	56.2	*β*-Amyrin	88	1.46
49	56.7	9,19-Cyclolanost-24-en-3-ol	75	0.41
50	57.7	Lupeol	84	5.16

**Table 4:** The results of the antimicrobial activity of *Portulaca oleracea*'s CO_2_ extract obtained by the serial dilution method.

Object of study	Minimum bactericidal concentration (*μ*g/ml)			
	*S. aureus* ATCC 6538-Р	*E. coli* ATCC 8739	*B. subtilis* ATCC 6633	*C. albicans* ATCC 10231
*Portulaca oleracea*'s CO_2_ extract	250	500	500	500

**Table 5:** The results of the antimicrobial activity of *Portulaca oleracea*'s CO_2_ extract obtained by the disco-diffuse method.

Object of investigation (*μ*g/ml)	Growth suppression zone (mm)			
	*S. aureus* ATCC 6538-Р	*E. coli* ATCC 8739	*B. subtilis* ATCC 6633	*C. albicans* ATCC 10231
*Portulaca oleracea*'s CO_2_ extract, 1000 *μ*g/ml	20.0	18.0	21.0	15.0

## Data Availability

The datasets used and/or analyzed during the current study are available from the corresponding author on reasonable request.
